# Surface defect detection on industrial drum rollers: Using enhanced YOLOv8n and structured light for accurate inspection

**DOI:** 10.1371/journal.pone.0316569

**Published:** 2025-02-05

**Authors:** Guofeng Qin, Qinkai Zou, Mengyan Li, Yi Deng, Peiwen Mi, Yongjian Zhu, Hao Liu

**Affiliations:** 1 Teachers College for Vocational and Technical Education, Guangxi Normal University, Guilin, People’s Republic of China; 2 Key Laboratory of Brain-inspired Computing and Intelligent Chips, School of Electronic and Information Engineering, Guangxi Normal University, Guilin, People’s Republic of China; 3 Guilin Normal College, Guilin, People’s Republic of China; 4 College of Engineering Physics, Shenzhen Technology University, Shenzhen, Guangdong Province, People’s Republic of China; Jamia Millia Islamia A Central University, INDIA

## Abstract

Drum roller surface defect detection is of great research significance for control production quality. Aiming at solving the problems that the traditional light source visual imaging system, which does not clearly reflect defect features, the defect detection efficiency is low, and the accuracy is not enough, this paper designs an image acquisition system based on line fringe structured light and proposes an improved deep learning network model based on YOLOv8n to achieve efficient detection of defects on the rolling surface of a drum roller. In the aspect of image acquisition, this paper selected the line fringe structured light as the system light source, which made up for the problem that the traditional light source does not reflect the defect characteristics. In terms of algorithms, firstly, using deformable convolution instead of standard convolution to enhance the feature extraction ability of the backbone network. Then, a new feature fusion module was proposed to enable the fusion network to learn additional original information. Finally, Wise-IoU was applied to replace CIoU in the loss function, so that the network pays more attention to the high-quality samples. The experimental results show that the improved YOLOv8n algorithm has a certain improvement in detection accuracy. The main average accuracy (mAP) is 97.2%, and the detection time is 4.3ms. The system and algorithm designed in this paper can better ensure the production quality of drum rollers. While effective, the model’s standard rectangular bounding boxes may limit precision for elongated defects. Future work could explore rotated bounding boxes and broader dataset diversity to enhance generalization in real-world applications.

## 1.Introduction

Bearings are the basic components for the transmission and operation of various mechanical devices, and widely used in fields such as robotics, aerospace, and automobile manufacturing. The drum roller is a common type of rolling bearing, and its rolling surface is the most susceptible to wear. Therefore, ensuring the quality of the rolling surface during production and assessing the degree of damage during use are crucial for the service life and safety of the equipment.

Drum rollers are a type of rolling bearing that are prone to wear, and the quality of their surfaces has a significant impact on the performance and lifespan of the equipment. Defect detection on the surfaces of bearing rollers is often implemented using machine vision, but traditional image processing algorithms used in machine vision suffer from low efficiency and are inadequate to meet the demands of modern industrial development. Currently, the most widely used method in industrial production is the defect detection technology based on machine vision, which has achieved a series of research results [1,2]. The traditional process of machine vision defect detection technology is to design an image acquisition system to acquire defect images, and then apply image processing algorithms to detect and identify defects. In the process of image acquisition, the light source directly affects the image quality, and the selection of the appropriate light source is crucial. Structured light is one of the early technologies applied in industrial defect detection, Huang et al [3] proposed a dust elimination defect detection method based on structured light modulation analysis, using polarized illumination and a linear polarizer to capture images. Subsequently, the traditional algorithm was used to analyze the image. Finally, the defect detection of mirror object was realized. Zheng et al [4] built an image acquisition system based on bimodal structured light sensors to collect intensity and depth images. Moreover, they proposed a parallel feature extraction deep learning model to complete rail surface defect detection and measurement, with mAP reaching 87.17% and a speed of 6.2 FPS on a self-built bimodal image dataset. Zoric et al [5] used Black Random Fringes Structured Light and Fringes Brown Light as the system light source to acquire images. A new Fourier spectrum annulus feature extraction method was proposed to achieve defect detection on ceramic tile surfaces, with F1 scores of 0.9236 and 0.8866 on the two light source datasets, respectively. Yuan et al [6] proposed a new spiral fringe structured light in response to the problems of small space for detecting small diameter inner wall surface reflective linear bearing sleeve and the difficulty of automatic detection, which realized micron defect detection and with 99.31% detection accuracy.

In the whole process of defect detection, designing efficient defect detection algorithms is as important as collecting defect images. The traditional algorithms such as edge detection and threshold segmentation, which are commonly used today, rely on manual feature extraction and tend to ignore useful information, and have the problem of low detection efficiency. In recent years, with the development of artificial intelligence, scholars have applied deep learning technology to the field of defect detection, which compensates for the shortcomings of traditional algorithms with its automatic feature extraction. The two-stage model represented by Faster RCNN, the one-stage model represented by YOLO series, and the deep learning model based on transformer have been widely applied. For example, Zheng et al [7] improved the Faster RCNN algorithm from two aspects of feature enhancement and prediction box aggregation. The improved algorithm not only reduces the missing detection caused by small objects, but also solves the multi box detection problem due to discontinuous objects. On the self-developed wafer dataset, the average accuracy of the improved model is 87.5%,and the detection time is 0.26s. Hu et al [8] built a new network based on Faster RCNN to achieve defect detection on the surface of printed circuit boards (PCBs), with mAP reaching 94.2% and detection time of 0.078s on a self-made dataset. Zhang et al [9] proposed an improved algorithm based on YOLOv5 to solve the problems of complex background and changeable defects of solar cell graphics. The average detection accuracy reached 89.64% and the speed reached 36FPS on the solar cell EL dataset. Xie et al [10] proposed a surface defect detection algorithm based on feature-enhanced YOLO (FE-YOLO) for practical industrial applications, achieving industrial surface defect detection. The effectiveness of the model was verified on NEU-DET and DeepPCB datasets, with defect detection accuracy of 83.9% and 98.9%, respectively. Gao et al [11] designed a new window transfer scheme to further strengthen the feature transfer between windows. The new window transfer scheme was introduced into Swin Transformer to obtain a new model Cas-VSwin, which achieved competitive results (82.3 box AP and 80.2 mask AP) on the private steel dataset. Wang et al [12] incorporated CNN and transformer into an efficient hybrid transformer architecture for defect detection, called Defect Transformer, which realized the detection of surface defects of different objects. The experimental results show that the detection effect of Defect Transformer is better than that of similar architecture models on the three datasets: SD-saliency-900 dataset, fabric defect dataset, and NRSD-MN dataset.

For drum rollers, the traditional light source cannot effectively highlight defect features due to their high reflectivity. In addition, the rolling surface defects of drum rollers contain a large number of small defects, and the current deep learning algorithms still have problems such as easy to miss detection and insufficient accuracy of small defect detection. Therefore, a suitable light source and an excellent algorithm are essential for the accurate detection of rolling surface defects. This paper selected the line fringe structured light to compensate for the shortcomings of traditional light sources that cannot fully reflect defect features. At the same time, efficient modules are introduced to improve the algorithm, which not only improves speed but also enhances detection accuracy. The main contributions of this work are as follows:

- This paper designed an image acquisition system for drum rolling surfaces based on line structured light, targeting the characteristics of drum rolling surfaces. Among them, the use of fringe structured light in the system improves the contrast of defect images and compensates for the shortcomings of traditional light sources. In addition, this paper designed a horizontal motion device as the transmission device of the system, eliminating the problem of motion blur. This system has completed image acquisition, providing a data foundation for subsequent algorithm research.
- Based on the YOLOv8n model, improved the backbone network. This paper introduces deformable convolution into the C2f module of the backbone network, replacing standard convolution, to enhance the feature extraction ability of the backbone network and enable the model to better learn defect characteristics.
- Based on the YOLOv8n model, the feature fusion module has been improved. This paper proposes the feature fusion module UpFPN, which adds a branch on top of the original feature fusion module, allowing the small object detection layer to learn additional raw information and improve the model’s ability to recognize small defects.
- Based on the YOLOv8n model, a more effective loss function has been introduced. This paper uses Wise-IoU instead of the original CIoU for regression loss calculation. Wise-IoU is a regression calculation based on distance attention mechanism, which effectively reduces the influence of geometric factors and can better fit annotation boxes. Effective regression loss can improve the accuracy of defect localization.

The rest of this paper is organized as follows. In section 2, the work related to the inspection of bearing surface defects is described. The details of the image acquisition system and the improvement of the detection network are presented in Section 3. Section 4 analyzes the experimental results. Section 5 summarizes the paper.

## 2.Related work

In recent years, a large number of excellent scientific research achievements have been made in the field of bearing surface defect detection. Feng et al [13] built an image acquisition system using point light source and annular light source, and proposed a prior model-guided network for defect detection. The network consisted of segmentation network, classification network, and pyramid feature fusion module, which improved the accuracy of bearing surface defect detection. The accuracy can reach 99.3% on the self-made dataset, but the model is complex and the detection efficiency is not high. Bao et al [14] designed a new image acquisition system based on strip light source and annular light source to complete the image acquisition of bearing cylindrical roller surface. Subsequently, traditional image processing algorithms such as threshold segmentation and blob analysis were used for defect detection, but there is a problem that features need to be extracted manually. Zhao et al [15] proposed a defect detection method consisting of region segmentation, feature extraction, and CNN detection network to complete the surface defect detection of universal joint bearings (UJB). The F1 score reached 94.8% and the speed reached 28FPS on the self-made dataset, but the traditional LED light source was used. Lu et al [16] proposed a bearing surface defect detection and classification algorithm based on machine vision. The local multi-neural network with wavelet transform as classification feature was used for image segmentation. In addition, the feature selection algorithm was improved. The defect detection rate reached 99.5%, but the detection efficiency of the algorithm is not high. Prappacher et al [17] proposed to use simulated defects for defect segmentation of bearing rollers, built an image acquisition system based on traditional LED light source to collect images to support training, and used convolutional neural network to realize defect image segmentation. However, the defects in the simulated data differed greatly from the real defects in terms of features, which had some influence on the detection results. Ling et al [18] proposed a lightweight bearing defect detection method based on depthwise separable convolution, which converts vibration signals into images as input. The detection accuracy reached 98.8% and 99% on the gearbox dataset of Southeast University and the bearing dataset of the CWRU (Case Western Reserve University), respectively. Nevertheless, such methods cannot determine the location of bearing defects. Yang et al [19] proposed an improved MobileNetV3 lightweight detection network, combined with fluorescent magnetic particle inspection technology, to achieve defect detection on the surface of bearing rings. The accuracy reached 96.5% on the self-made dataset, but the detection time was 9.33s, so the detection efficiency was low. Wu et al [20] proposed an automatic bearing inner ring defect detection system to collect images. Then, image processing and pattern recognition methods was used to detect defects. The recognition rate was 98.6%, and the detection time was about 1-2s. Xu et al [21] designed an unsupervised neural network for defect detection of bearing surface, built a device based on a ring LED lighting system for defect image acquisition, and realized the defect detection of bearing surface. The model AUC reached 97.21%.

The above research results show that most researchers do not focus on acquiring high-quality defect images, and researchers prefer to find efficient algorithms to improve the accuracy of defect detection. However, image quality limits the upper limit of defect detection, high-quality defect images are the basis of defect detection, and excellent algorithms are the keys to defect detection. Therefore, this paper improves both image acquisition and detection algorithms to improve the accuracy and speed of defect detection on the rolling surface of drum rollers.

## 3.Proposed method

### 3.1Image acquisition system

**Fig 1:**
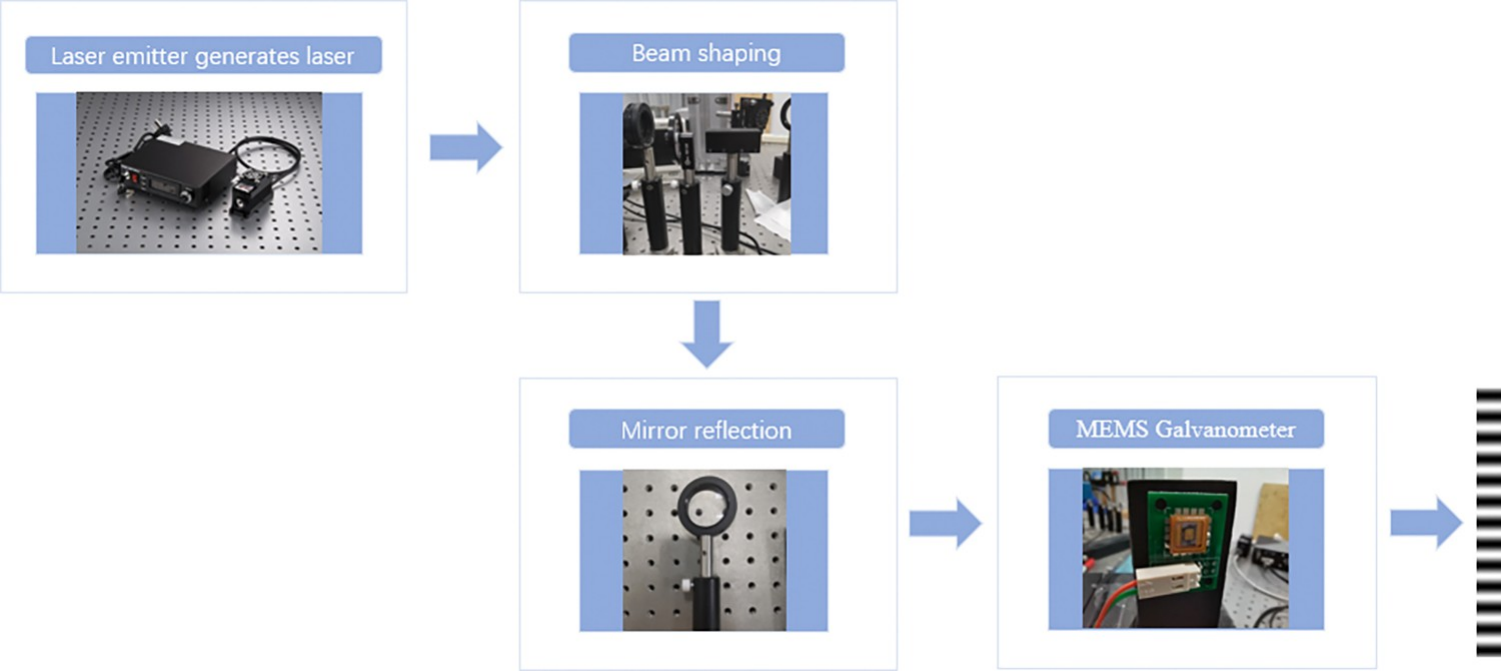
Line fringe structured light generation process.

The image acquisition system consists of light source, camera, lens, and mechanical motion system. Among them, the suitable light source is the basis of imaging. In view of the problem of insufficient reflection of defect features by traditional light source, in this paper, we choose line fringe structured light as the system light source. Line fringe structured light is produced by laser modulation. First of all, the signal generator controls the laser emitter to generate sinusoidal laser. Then, the laser filters the stray light through the stop, and the attenuator adjusts the brightness. Thirdly, the mirror reflects the laser into the MEMS galvanometer. Finally, the laser incident into the MEMS galvanometer generates line fringe structured light. The process is shown in Fig 1.

**Fig 2:**
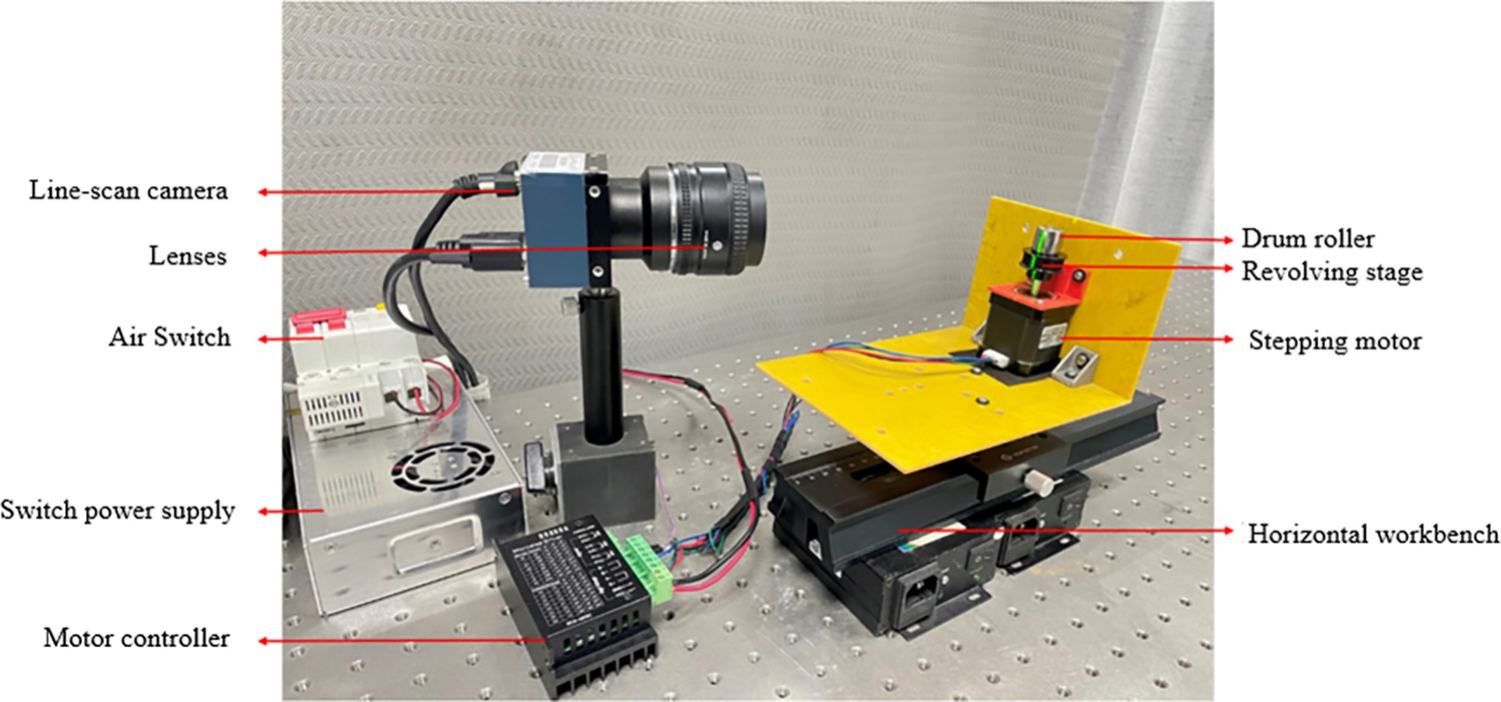
Mechanical motion device.

In addition, we need to capture the complete image of the drum roller rolling surface, so the motion device is key to the system. Therefore, we designed a mechanical motion device based on a horizontal turntable to synchronize the drum roller with the turntable. This device avoids the problem of image blurring caused by the distance generated by the relative motion between the drum roller and the motion device. The mechanical motion device is shown in Fig 2.

**Fig 3:**
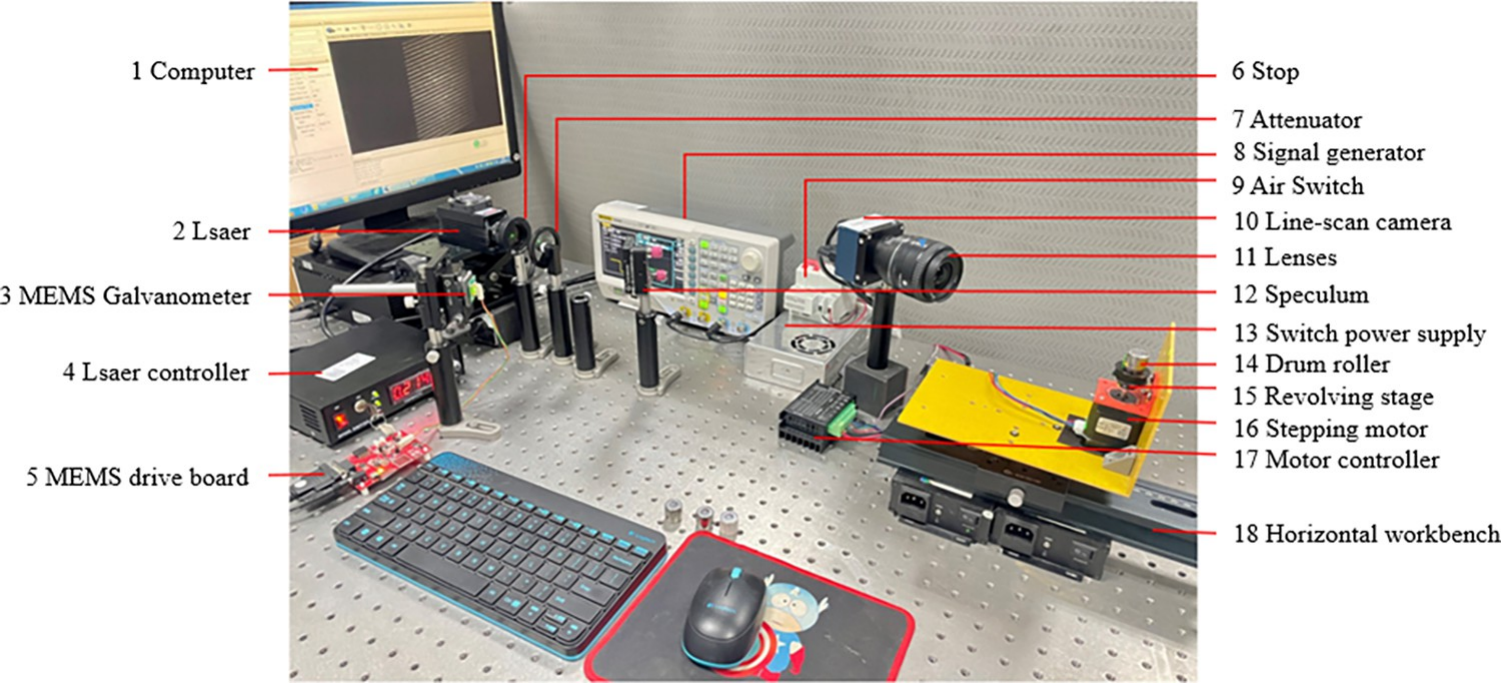
Linear fringe structured light image acquisition system.

On this basis, we select the appropriate camera, lens and other hardware equipment to build the drum roller rolling surface image acquisition system based on line fringe structured light. The physical diagram of the system is shown in Fig 3, which includes mechanical motion device, light source scanning device, and image acquisition device.

**Fig 4:**
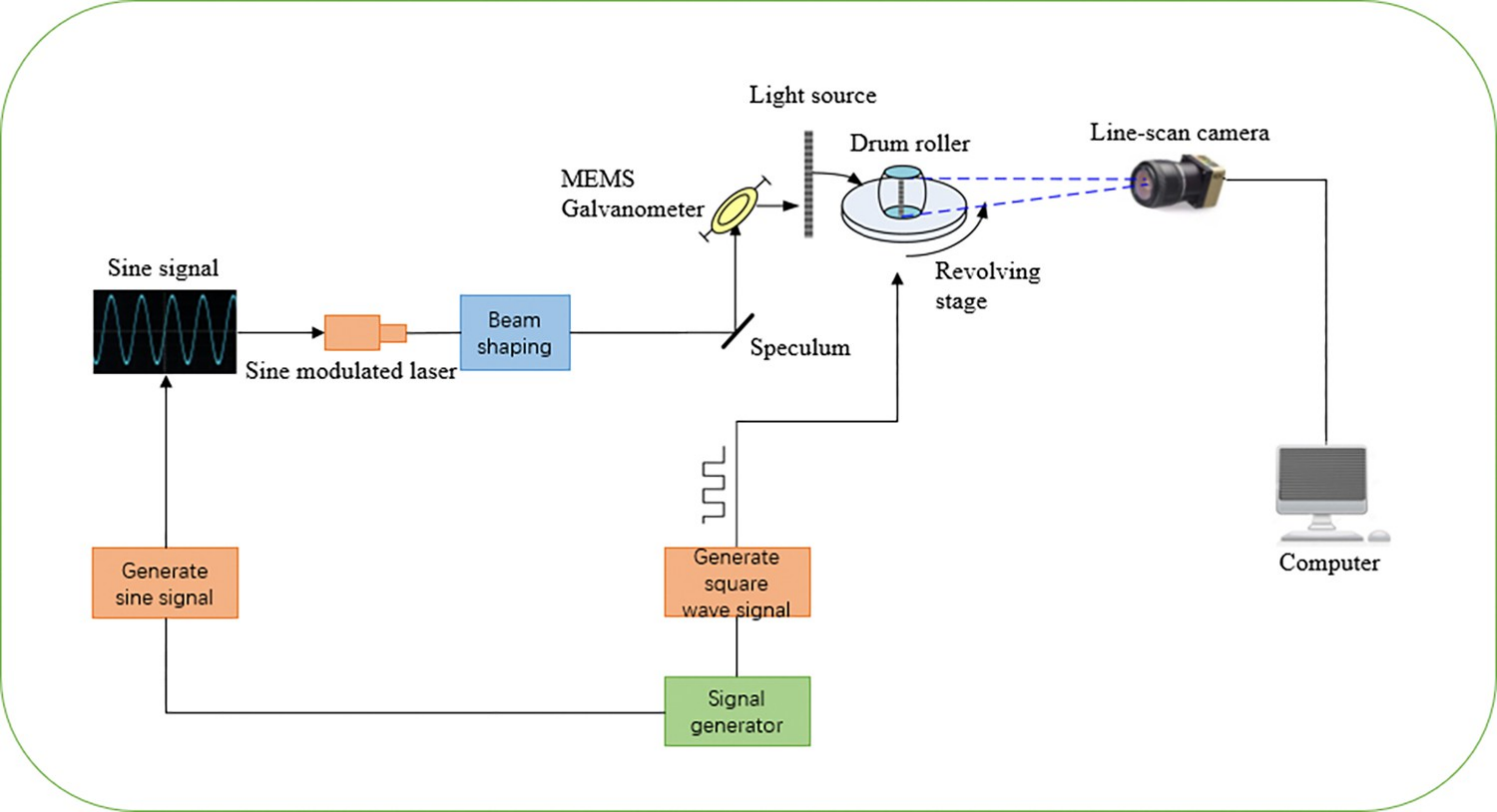
System working principle.

**Fig 5:**
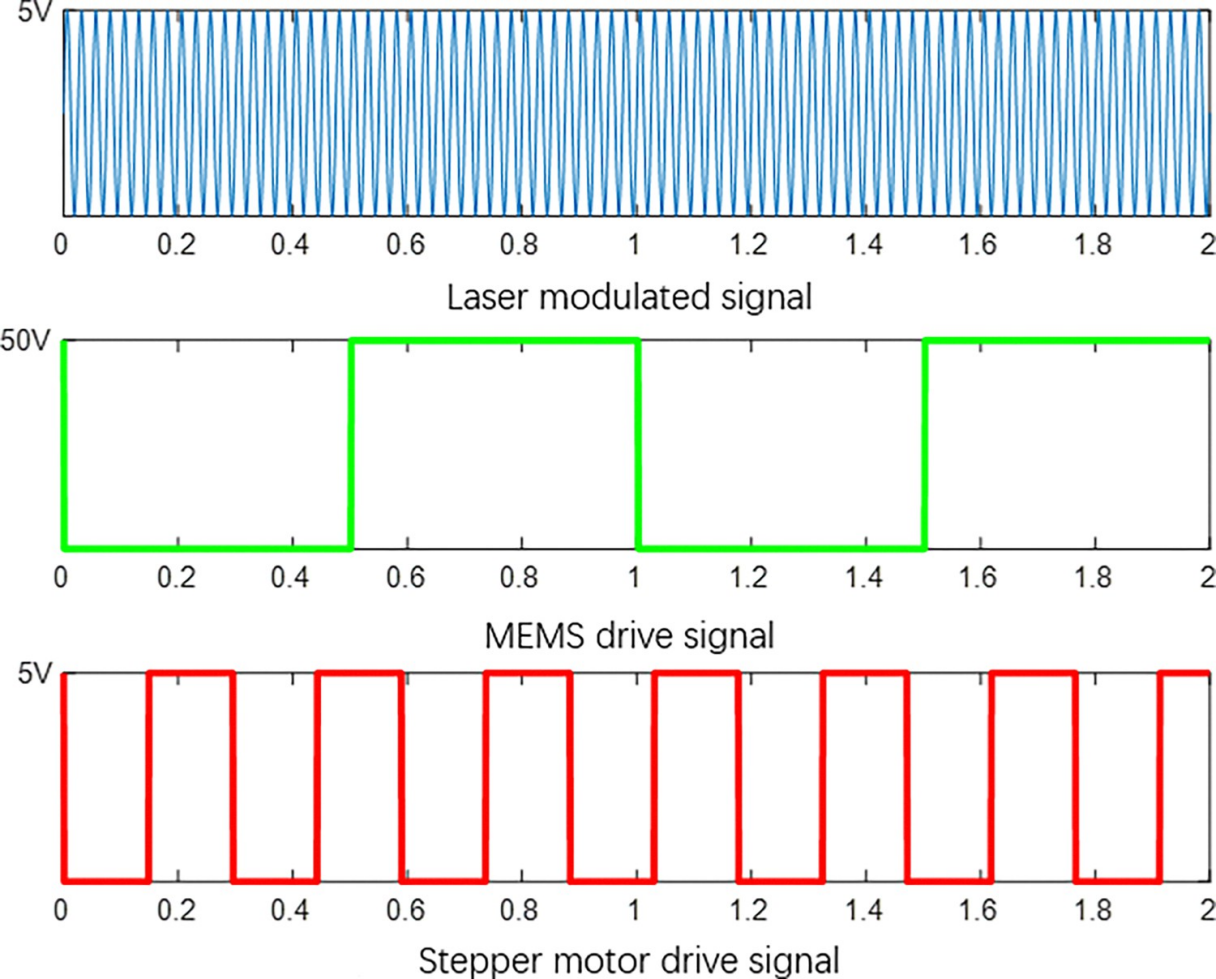
System signal.

In the entire system, the generation of light sources and the movement of mechanical devices rely on signal driving. The normal operation of the system depends on the synchronization of driving signals. The system working principle is shown in Fig 4. This paper selected appropriate MEMS driving signals, motor driving signals, and laser modulation signals through experimental testing to achieve system imaging. The motor drive signal is a square wave signal with a frequency of 3.4Khz, low-level 0v, and high-level 5v. The laser modulation signal is a sinusoidal signal with a frequency of 45Khz, a low point voltage of 0v, and a high point voltage of 5v. The MEMS galvanometer driving signal is a square wave signal with a frequency of 999hz, a low level of 0v, and a high level of 50v. Each signal is shown in Fig 5, with the unit of ms.

**Fig 6:**
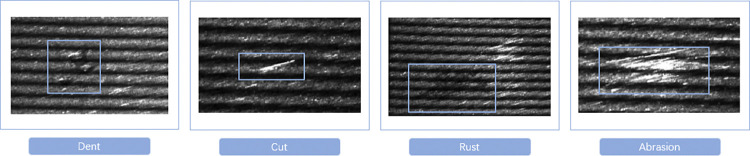
Four types of surface defects.

Through the image acquisition system of drum roller rolling surface based on line fringe structured light, we collected the defect image of drum roller rolling surface with good quality. Among them, there are mainly four types of injuries: Dent, Cut, Rust, and Abrasion, as shown in Fig 6.

### 3.2Detection network

YOLOv8 is the latest version of the YOLO series of networks, which integrates the latest research results for improvement and update, greatly improving the performance of tasks such as object detection, image segmentation, and image classification. The YOLOv8 model is divided into five different scales, it controls the depth and width of the model by adjusting parameters, so that the model can adapt to different equipment conditions. Among all scales of YOLOv8, YOLOv8n is the smallest model, and has good detection accuracy, the least number of parameters, and the lowest computational cost. Therefore, this paper chooses the YOLOv8n model as the baseline, and improves it from the aspects of backbone network, loss function, and feature fusion network.

**Fig 7:**
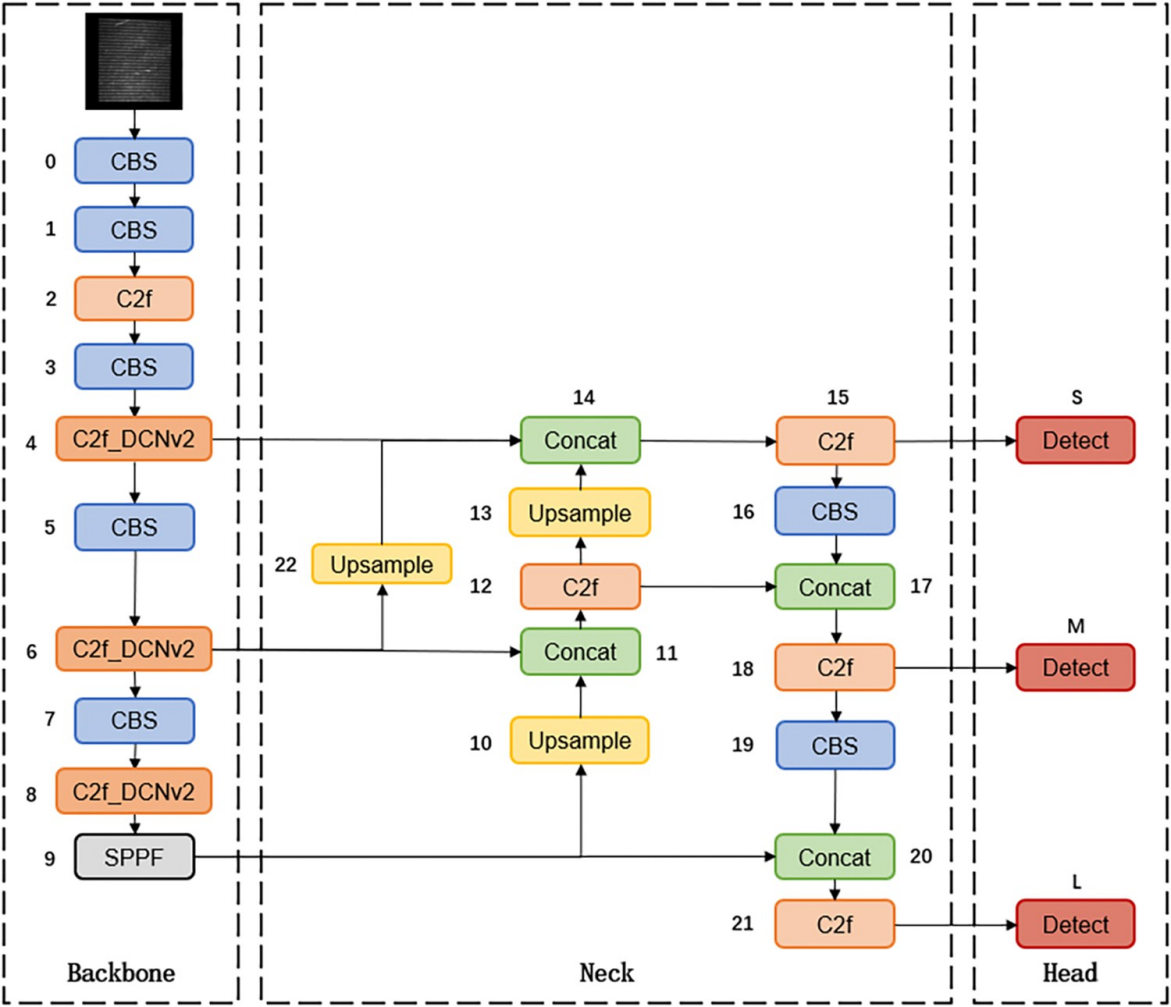
Overall structure of the model.

The overall structure of the improved model is shown in the following Fig 7, and the training process can be divided into four parts: input, backbone network, feature fusion, and prediction output. The task of the input stage is to perform some preprocessing operations on the input images before training. The backbone network is responsible for extracting features from the input images. The feature fusion part ensures that both deep and shallow information are effectively integrated, while the prediction output provides the location and category of the defects.

#### 3.2.1 Deformable convolution

The backbone network for feature extraction is based on the convolution operation, and standard convolution is used in YOLOv8n. The perceptual field of the standard convolution is fixed at the same position, however, the constant perceptual is not conducive to adjusting the shape and size of the object during feature extraction. Therefore, this paper introduces the more flexible deformable convolution [22]. Deformable convolution is used to guide the convolution kernel to offset according to the characteristics of the object features by convolving the feature map with the convolution layer. The shifted convolutional kernel is more approximate to the shape of the detected object. In addition, the convolution that generates the offset and the convolution that extracts the features are learned simultaneously during the training process.

Due to the computational complexity of deformable convolution, this paper primarily uses deformable convolution in the bottleneck of the last three C2f modules of the backbone network. The C2f module is the most important part of the backbone network, which uses parallel Bottleneck branches to obtain richer gradient information to improve the performance of the model. The details of the deformable convolution improvement are shown in Fig 7, where (a) is the C2f model structure, (b) is the original Bottleneck structure, and (c) is the DCNv2-Bottleneck structure.

#### 3.2.2 Loss function

YOLOv8 classification loss uses VFL loss, which introduces an asymmetric weighting operation to highlight the main positive sample. The regression loss uses CIoU Loss combined with DFL. Among them, DFL establishes a general distribution of the bounding box to increase the probability that the network focuses on the object. CIoU loss is used to calculate the bounding box loss, which is defined as follows:

$$CIoU=IoU-\frac{\rho^{2}(b,b_{gt})}{c^{2}}-\alpha v$$ (1)

$$\alpha =\frac{v}{(1-IoU)+v}$$ (2)

$$v=\frac{4}{\pi^{2}}{\left(arctan\frac{w^{gt}}{h^{gt}}-arctan\frac{w}{h}\right)}^{2}$$ (3)

$$L_{CIoU}=1-CIoU$$ (4)

CIoU loss defines the parameter *v* to account for the effect of the bounding box aspect ratio. As shown in Eq (3), where $\frac{\partial v}{\partial h}=-\frac{w}{h}\frac{\partial v}{\partial w}$, indicating that *v* provides opposite gradients for the width and height of the anchor, which would cause the model to fail to regress to the optimal bounding box.

**Fig 8:**
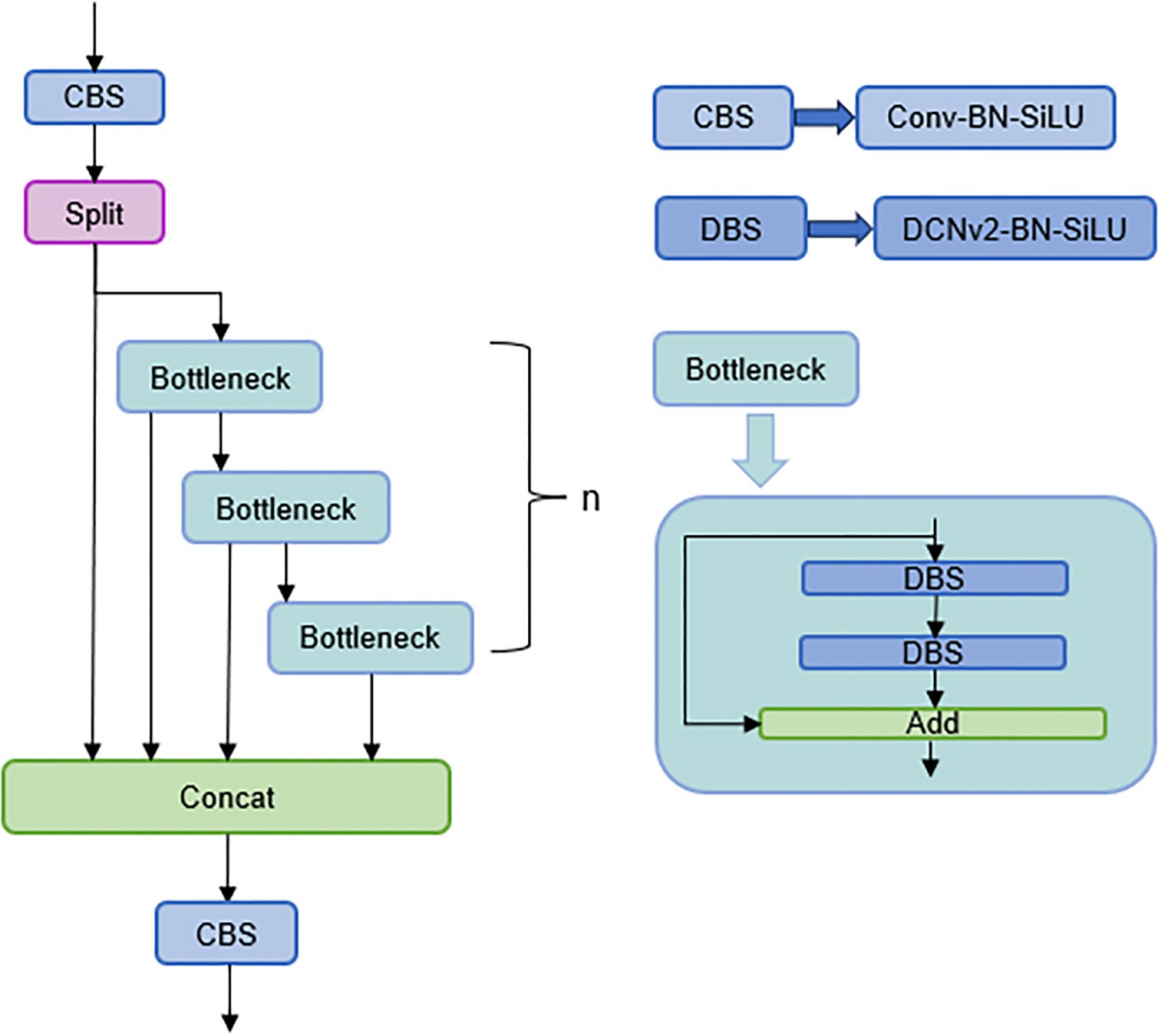
Deformable convolution improvement details.

Since there are inevitably low-quality samples in the dataset, the choice of the penalty basis of the loss function has a great impact on the generalization ability of the model. However, the geometric factors such as center distance and aspect ratio are more likely to focus on the low-quality samples. Therefore, in this paper, Wise-IoU v3 Loss (WIoU v3) [23] is chosen as the loss function of the bounding box. Based on the distance attention mechanism, it can not only reduce the influence of geometric factors, but also better fit the bounding box. The meanings of the letters in the formula are shown in Fig 8, and the formula is defined as follows:

$$L_{IoU}=1-IoU=1-\frac{w_{i}h_{i}}{wh+w_{gt}h_{gt}-w_{i}h_{i}}$$ (5)

$$L_{WIoUv1}=R_{WIoU}L_{IoU}$$ (6)

$$R_{WIoU}=exp(\frac{{\left(x-x_{gt}\right)}^{2}+{\left(y-y_{gt}\right)}^{2}}{{\left(w_{u}^{2}+h_{u}^{2}\right)}^{*}})$$ (7)

$$L_{WIoUv3}=rL_{WIoUv1},r=\frac{\beta }{\delta \alpha^{\beta -\delta }},\beta =\frac{L_{IoU}^{*}}{\bar{L_{IoU}}}$$ (8)

In Eq (8), $\bar{L_{IoU}}$ is the running average of the momentum m. The default value of m is $1-\sqrt[{7000}]{0.5}$, the default value of *α* is 1.9, the default value of *δ* is 3.

#### 3.2.3 Feature fusion network

**Fig 9:**
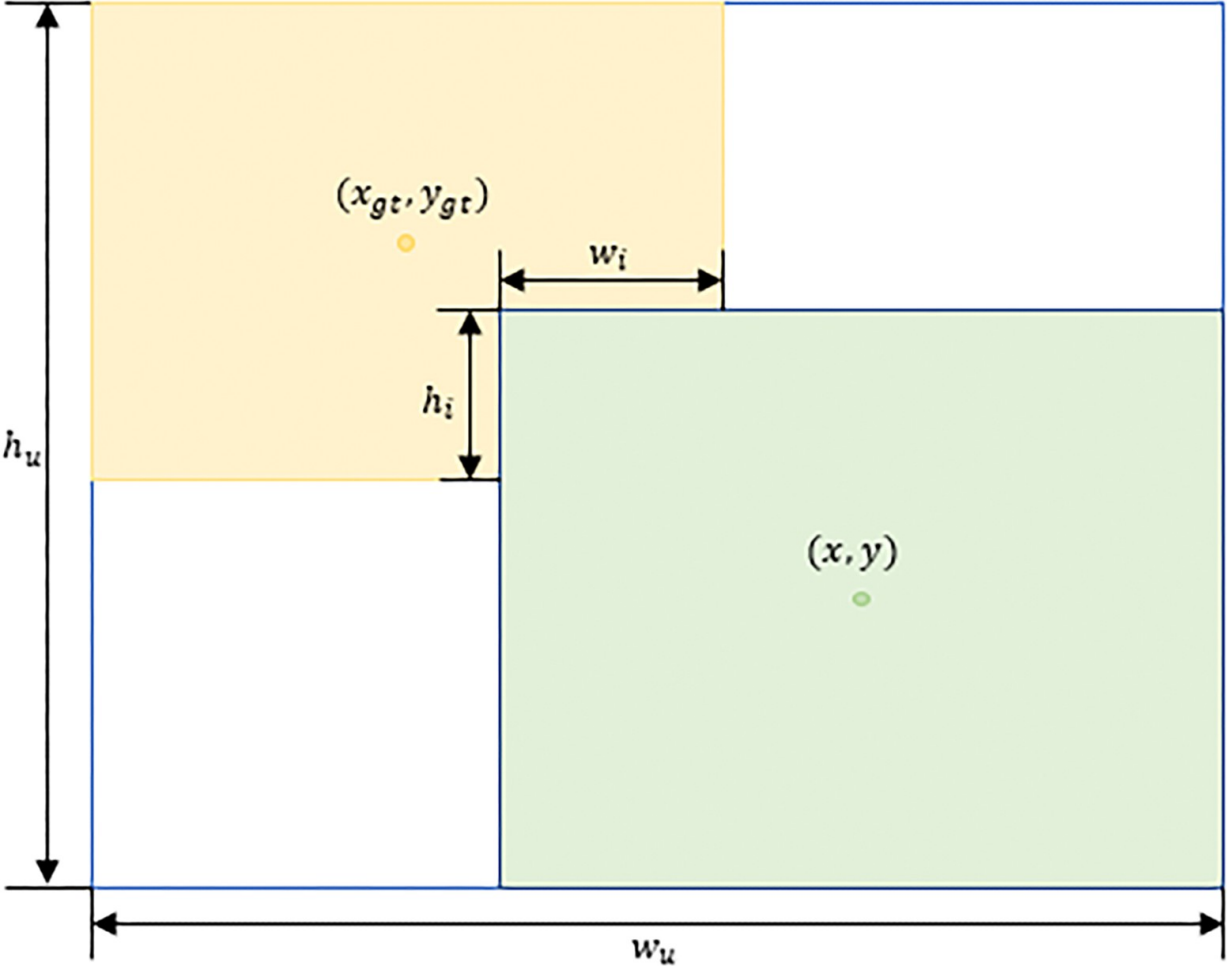
IoU.

**Fig 10:**
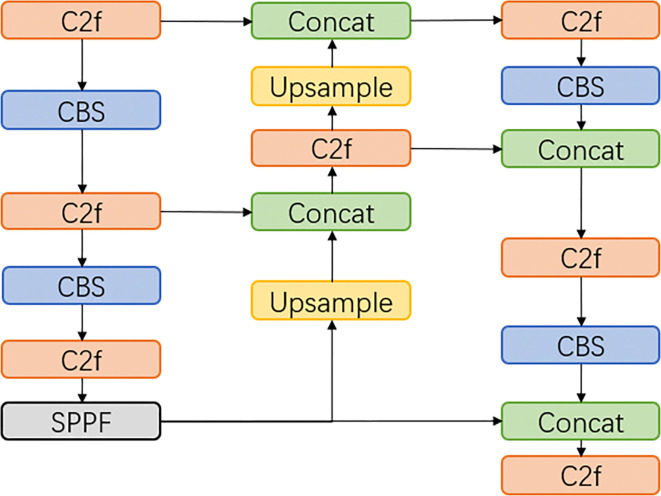
PAN-FPN model structure.

**Fig 11:**
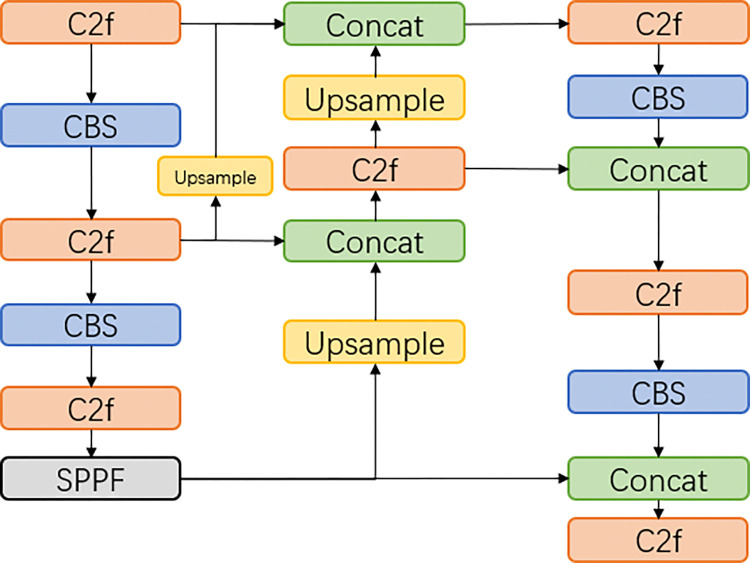
UpFPN model structure.

The neck of YOLOv8 uses the structure of PAN-FPN [24], as shown in Fig 9. Although PAN-FPN has information fusion in both top-down and bottom-up directions, the medium-scale information is combined with the features of the small-scale detection layer only after the convolution operation, which leads to partial information loss. Therefore, this paper adds a new branch to PAN-FPN and designs a new feature fusion structure, UpFPN. It is implemented by taking out the output of the penultimate C2f layer in the backbone network, and then changing the size of the feature map by up-sampling the layer, using the nearest-neighbor interpolation method, the up-sampled output features are concatenated with the output of the small object detection layer. UpFPN enables the network to additionally learn more original image features, reduce information loss, and enhance feature fusion, thus improving the detection accuracy of the network. The detailed structure of PAN-FPN and UpFPN are shown in Figs 10 and 11.

## 4.Experiment

### 4.1Dataset

**Fig 12:**
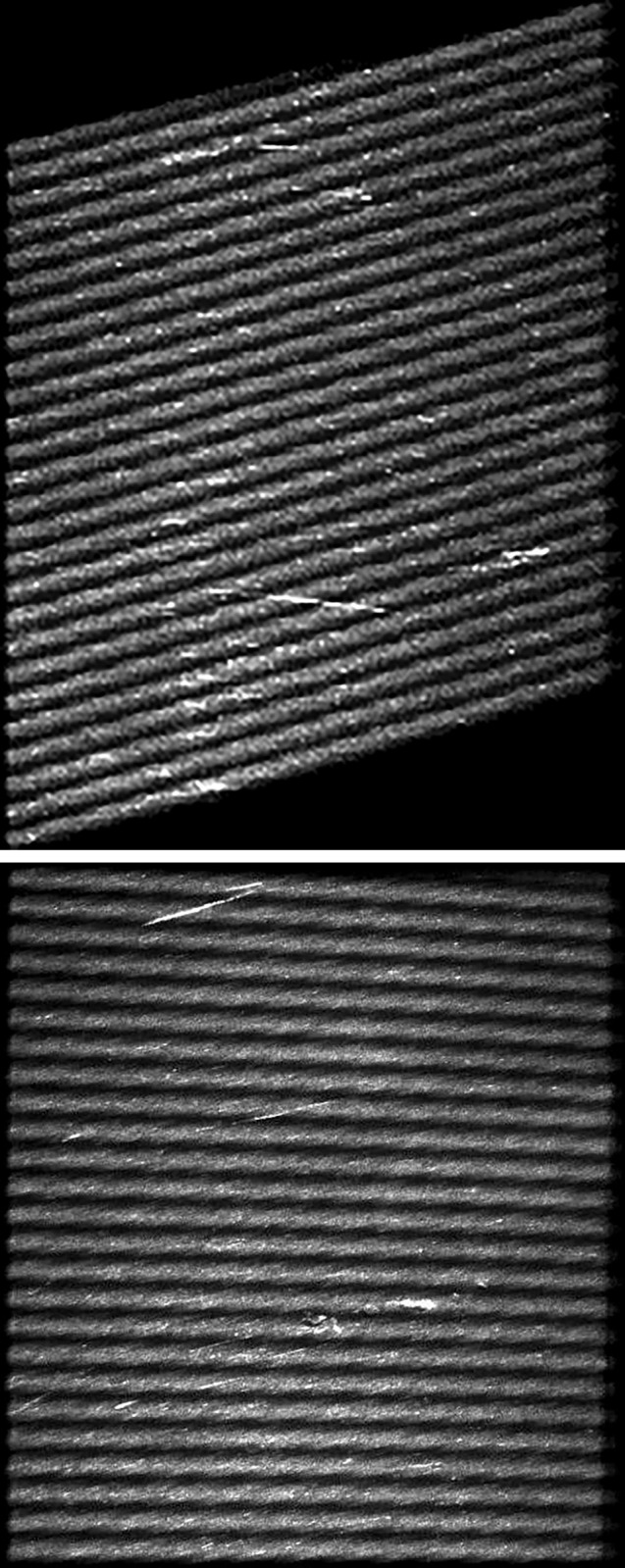
Image cropping. (a) original image. (b) cropped image.

This paper uses an image acquisition system based on line fringe structured light to collect 558 original images of drum roller rolling surface. During the image acquisition process, it is challenging to ensure that the drum roller and the camera’s field of view are perfectly vertical or horizontal. Additionally, it is difficult to achieve absolute parallelism between the scanning direction of the structured line fringe light and the motion direction of the drum roller. As a result, the original images captured by the system exhibit fringes in the shape of parallelograms with relative displacement, and the original size is 1080×1024 pixels. The acquired original image is shown in Fig 12A. Due to the tilt of the fringes in the original image, it is necessary to perform tilt correction before constructing the defect detection dataset. This correction transforms the tilted parallelogram into a rectangular region through affine transformation. Affine transformation is a mapping process between images, represented as the multiplication of the original matrix by a linear transformation matrix, followed by the addition of a translation vector. Since the original image contains black regions with no information, a rectangular bounding box was selected using pixel point positioning to crop the image. After cropping, the image size was reduced to 640×640 pixels. The corrected and cropped image is shown in Fig 12B. Due to the small amount of data, this paper uses random offline data augmentation to expand the dataset, mainly including random cropping, adding noise, dynamic blurring and other enhancement methods. After augmentation, 2000 images are available, which are randomly divided into training set and validation set in the ratio of 8:2, and the drum roller rolling surface line fringe structured light dataset (DRSD) is constructed.

### 4.2 Model training

Experimental hardware devices: Intel(R) Xeon(R) E5-2630 v3 CPU, NVIDIA GeForce RTX 3080 GPU, Ubuntu 18.04 OS, PyTorch framework. Hyperparameter settings: batchsize is set to 16, epochs to 300, optimizer to SGD, initial learning rate to 0.01, and weight decay parameter set to 0.0005.

### 4.3 Evaluation metrics

In this paper, the average mean accuracy with IoU threshold of 0.5 (mAP50), the average mean accuracy with IoU threshold of 0.5 to 0.95 steps of 0.5 (mAP50-95). The mAP50 refers to the mean Average Precision (mAP) at a 50% IoU (Intersection over Union) threshold, meaning that if the overlap between the predicted and ground truth bounding boxes reaches 50%, the detection is considered correct. The mAP50-95, on the other hand, represents the average precision of the model calculated at various IoU thresholds (typically ranging from 0.5 to 0.95, with a step size of 0.05), and then averaging the precision scores across these thresholds. The number of model parameters (Params), the total model computations (FLOPs), and the model inference time (Time) are used as the evaluation metrics of the model. The formula is defined as follows:

$$P=\frac{TP}{TP+FP}$$ (9)

$$R=\frac{TP}{TP+FN}$$ (10)

$$AP=\int_{0}^{1}PdR$$ (11)

$$mAP=\frac{\sum_{i=1}^{N}{AP}_{i}}{N}$$ (12)

Where TP is the number of correctly predicted objects, FP is the number of incorrectly predicted objects, FN is the number of missed objects, and mAP50-95 is a more stringent metric for evaluation than mAP50.

### 4.4 Ablation experiment

**Fig 13:**
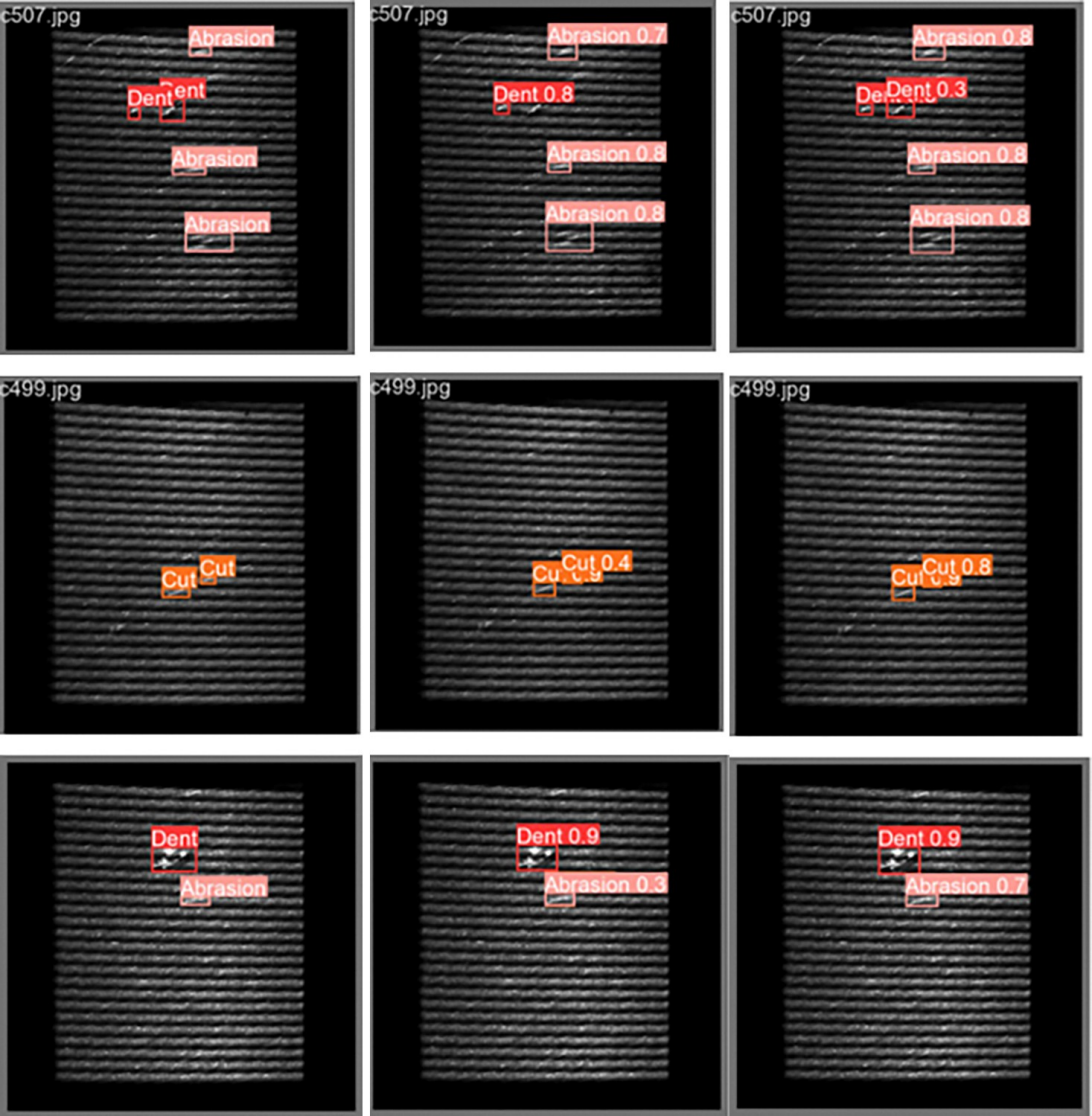
Defect detection results. (a) labels. (b) YOLOv8n. (c) YOLOv8n-wdn.

In this paper, YOLOv8n is trained on the DRSD dataset as the baseline model. By gradually superimposed improvement points, we verified the effectiveness of the above three improvement points. The hyperparameters and experimental conditions were kept consistent during the experiment. The evaluation metrics results are shown in Table 1. The defect detection results, shown in Fig 13, demonstrate the significant improvements of the YOLOv8n-wdn model compared to the baseline. Specifically, YOLOv8n-wdn increases mAP50 by 1.2% and mAP50-95 by 1.9%, while the number of parameters only increases by 0.16M, the computational volume decreases by 0.5G, and the inference speed improves by 0.2ms. These improvements are attributed to several key factors. First, the introduction of deformable convolution allows the network to adaptively adjust its receptive field, enhancing its ability to detect defects of varying shapes and sizes, especially on reflective surfaces where traditional convolutional methods often fail. This flexibility is crucial for accurately identifying irregular and small defects, leading to higher overall accuracy. Second, the replacement of CIoU with Wise-IoU in the loss function allows the model to focus more on high-quality samples, improving the precision of bounding box regression and contributing to the increase in mAP50-95. Finally, the inclusion of the UpFPN feature fusion module ensures that deep and shallow feature information is better integrated, significantly improving the detection of small defects that are often missed. These architectural improvements not only enhance accuracy but also maintain the model’s computational efficiency, making it well-suited for real-time industrial applications.

**Table 1:** Comparison of model evaluation metrics.

*Model*	*Wise-IoU*	*DCNv2*	*UpFPN*	*mAP50*	*mAP50-95*	*Params*	*FLOPs*	*Time*
YOLOv8n				95.9%	74.2%	3.01M	8.9G	4.5ms
YOLOv8n-w	√			96.7%	75.3%	3.01M	8.9G	4.1ms
YOLOv8n-wd	√	√		97.3%	75.6%	3.16M	8.3G	4.4ms
YOLOv8n-wdn	√	√	√	97.1%	76.1%	3.17M	8.4G	4.3ms

To better evaluate the model’s performance and ensure its generalizability, a five-fold tests [25] was employed for validation. As shown in Table 2, the proposed method outperforms YOLOv8n in both mAP50 and mAP50-95 metrics across the five-fold tests, demonstrating enhanced stability.

**Table 2:** Five-fold tests.

	YOLOv8n (mAP50/ mAP50-95)	YOLOv8n-wdn (mAP50/ mAP50-95)
Fold 1	95.9%/74.2%	97.2%/76.1%
Fold 2	95.6%/74.0%	97.0%/75.8%
Fold 3	96.1%/74.4%	97.4%/76.5%
Fold 4	96.0%/74.5%	97.3%/76.2%
Fold 5	95.7%/73.9%	97.1%/76.0%
Avg	95.86%/74.18%	97.2%/76.12%

### 4.5 Comparative experiment

In order to further demonstrate the validity of the model, this paper adds the cross-sectional comparative experiment of popular models. We select the classical two-stage network Faster-RCNN [26], the classical single-stage anchor-free network FCOS [27], the most widely used one-stage object detection network YOLOv5s, and the network YOLOv8s of different scales in the YOLOv8 series as the comparison model. The comparison results are shown in Table 3. In addition, this article also conducts defect detection comparative experiments using traditional image processing algorithms. Traditional algorithms predict defect areas through Blob analysis after segmentation algorithms and morphological processing. Traditional algorithms measure the accuracy of defect detection at the image level, and if a defect is detected, it is judged as a defective product. In the sample set, the accuracy of defect sample prediction reached 96.9%. However, traditional algorithms cannot predict defect types and have slow detection speed, with an average processing time of 1053ms per image. The experimental data show that the improved model not only has a better trade-off in accuracy and speed, but also can meet the needs of drum roller rolling surface defect detection. It can be seen that the improved model has practical value.

**Table 3:** Model cross-sectional comparison.

*Model*	*mAP50*	*mAP50-95*	*Params*	*FLOPs*	*Time*
Faster-RCNN	83.4%	-	41.37M	134.51G	32.4ms
FCOS	93.7%	-	32.12M	80.6G	45ms
YOLOv5s	96%	74.3%	6.7M	15.8G	13.2ms
YOLOv8n	95.9%	74.2%	3.01M	8.9G	4.5ms
YOLOv8s	97.7%	78.4%	10.65M	28.8G	5.6ms
YOLOv8n-wdn(ours)	97.1%	76.1%	3.17M	8.4G	4.3ms

The model configuration used in the experiment is as follows:

- Faster-RCNN:

Backbone: ResNet-50

Detection head: Region Proposal Network (RPN)

Anchor settings: 3 scales (32, 64, 128), 3 aspect ratios (0.5, 1, 2)

RoI Pooling: Yes

- FCOS:

Backbone: ResNet-50-FPN

Detection head: Fully convolutional head with centerness and regression branches

Anchor-free model: No anchor settings required

- YOLOv5s:

Backbone: CSPDarknet53

Detection head: PANet-FPN

Anchor settings: Default anchors (3 per scale)

The improvements in detection accuracy, particularly the 1.2% increase in mAP50 and 1.9% in mAP50-95, can be attributed to several key factors. First, the introduction of deformable convolution enabled the network to better capture irregular defect shapes, especially on reflective surfaces, by dynamically adjusting the receptive field. Second, the use of Wise-IoU in the loss function allowed the model to prioritize high-quality samples, leading to more accurate localization of defects. Finally, the UpFPN module ensured better fusion of deep and shallow features, improving the detection of small defects without increasing computational complexity. These enhancements contributed to both higher accuracy and faster inference, making the model suitable for real-time industrial applications.

In this study, although larger models such as YOLOv8s, YOLOv8x [28] may offer slightly higher detection accuracy, they significantly increase the number of parameters and computational complexity, making them unsuitable for industrial scenarios that demand real-time performance and limited computational resources.

## 5. Conclusion

In this study, we designed a line fringe structured light image acquisition system tailored for drum roller surface defect detection, significantly improving defect image contrast. Combined with improvements to the YOLOv8n network—enhancing the backbone, feature fusion, and loss function—the resulting YOLOv8n-wdn model achieved a mAP50 of 97.1%, mAP50-95 of 76.1%, and a detection time of 4.3ms, offering both higher accuracy and speed compared to the original network.

The study presents two significant strengths. First, the line fringe structured light-based acquisition system enhances the contrast of defect images, thereby providing a high-quality data foundation for defect detection. Second, the improved YOLOv8n-based algorithm not only increases the accuracy of detecting surface defects but does so without sacrificing speed, making it ideal for real-time industrial applications.

One limitation of the current defect detection algorithm is the use of standard rectangular bounding boxes, which may not effectively capture elongated or tilted defects like scratches, often including excessive background and reducing localization precision. Future work could explore the use of rotated bounding boxes to better match defect shapes and minimize irrelevant background. Additionally, the model’s generalization is limited by a lack of diversity in the dataset; expanding it to include a wider range of defect types and surface conditions could enhance performance in real-world scenarios. This represents an important area for future research.
